# Idiopathic Optic Nerve Glioma: A Case Report

**DOI:** 10.7759/cureus.76112

**Published:** 2024-12-21

**Authors:** Megha R Kotecha, Varsha D Singh, Surbhi A Chodvadiya, Varsha Manade, Gufran A Kamdar

**Affiliations:** 1 Department of Ophthalmology, Dr. D. Y. Patil Medical College, Hospital and Research Centre, Pune, IND

**Keywords:** neurofibromatosis 1, optic nerve atrophy, optic nerve glioma, pilocytic astrocytoma, tumors

## Abstract

Optic nerve gliomas are tumors that develop along the optic nerve pathway, most often classified as pilocytic astrocytomas. These growths are typically benign, especially in young children between the ages of one and six years, while the rarer malignant types are generally more aggressive and tend to appear in adults. Characteristically slow-growing, optic nerve gliomas are commonly located in the pre-chiasmal part of the optic nerve but can extend to post-chiasmal regions and into the brain if left untreated. We describe a case of a one-year-old boy who visited our ophthalmology clinic with symptoms of eye prominence and deviation in his right eye after undergoing chemotherapy. Clinical findings and additional assessments supported an optic nerve glioma diagnosis. This case underlines the significance of early detection and a collaborative approach among ophthalmologists, neurosurgeons, radiologists, and histopathologists for effective management and to limit disease progression.

## Introduction

The 1.2 million axons that make up the optic nerve are responsible for vision [1]. The most commonly occurring tumors of the optic nerve are optic nerve gliomas (ONG). Typically polycystic tumors mostly affect the optic nerve or the optic chiasm. ONGs are associated with type 1 neurofibromatosis (NF1) in 30% of cases, and are rare, slowly progressing benign tumors [2]. Usually unilateral, they occur most commonly in females [3]. They most commonly occur in the first 20 years of life, with 6.5 years being the median age and 10.9 years as the mean age, but they can occur at any age [4,5]. Symptoms and signs that ONGs present with are as follows: proptosis and often associated with inferior displacement of the globe, reduced vision, strabismus, and optic disc edema or pallor. In typical presentation ONGs, there is usually no ocular pain. Owing to persistent compression of the central retinal vein, a small number of individuals with ONGs may develop venous stasis retinopathy, rubeosis iridis with neovascular glaucoma, optociliary shunt vessels, and central retinal vein occlusion (CRVO). Additionally, sudden vision loss as well as the onset or exacerbation of proptosis are possible side effects. Some ONGs are asymptomatic, while partial or total loss of vision may be revealed on neurological examination [6,7]. Additionally, increased intracranial pressure may be present. Radiological investigations like CT-orbit scans, MRI-brain, and biopsies are used in the diagnosis of ONGs [8]. All treatment methods strive to preserve vision for as long as feasible. Treatment options for ONGs include surgery and radiation.

## Case presentation

**Figure 1:**
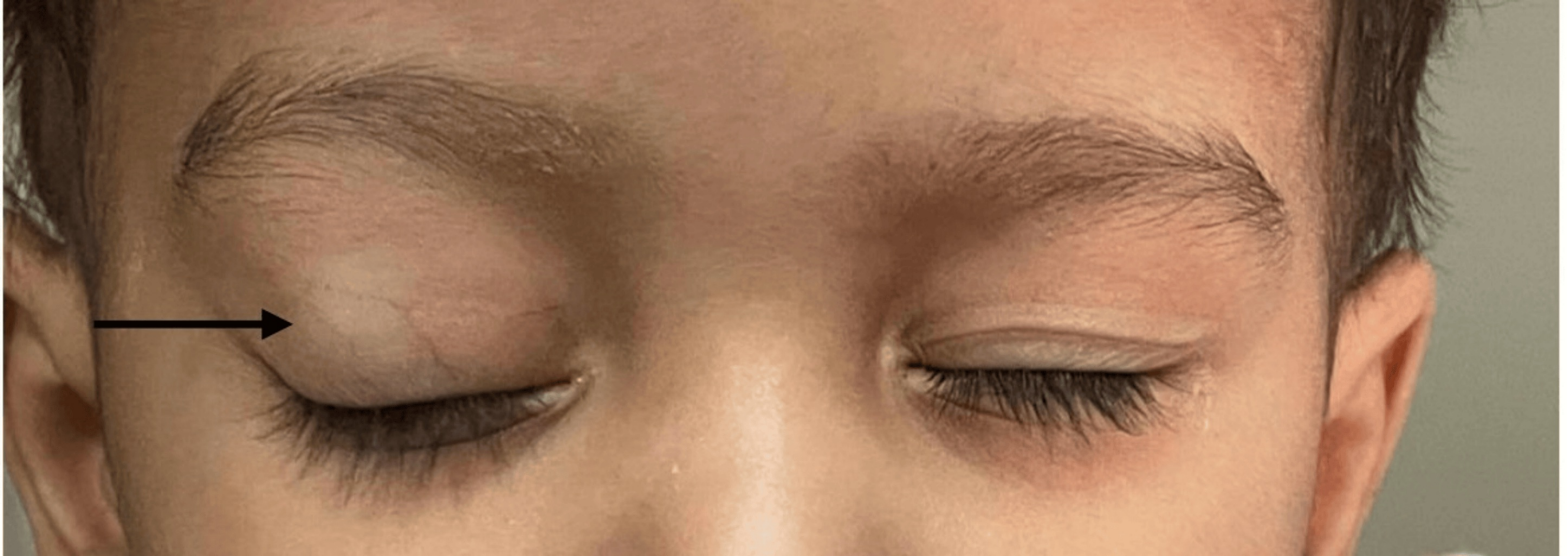
Proptosis of the right eye marked by black arrow.

We report a case of a one-year-old male child, whose reliable informant were his parents, who came to our OPD with chief complaints of prominence and deviation of his right eye. He had a history of receiving seven cycles of chemotherapy (vincristine). The patient was born to non-consanguineous parents and has a two-year-old elder brother, who had no ocular complaints. On a detailed and comprehensive ocular examination of the patient, the right eye revealed no perception of light. Pupillary examination showed a mid-dilated pupil, non-reacting to light. Proptosis was also noted (Figure 1). Posterior segment examination revealed generalized optic disc pallor and venous tortuosity, while no other retinal signs were seen. The left eye examination was within normal limits.

**Figure 2:**
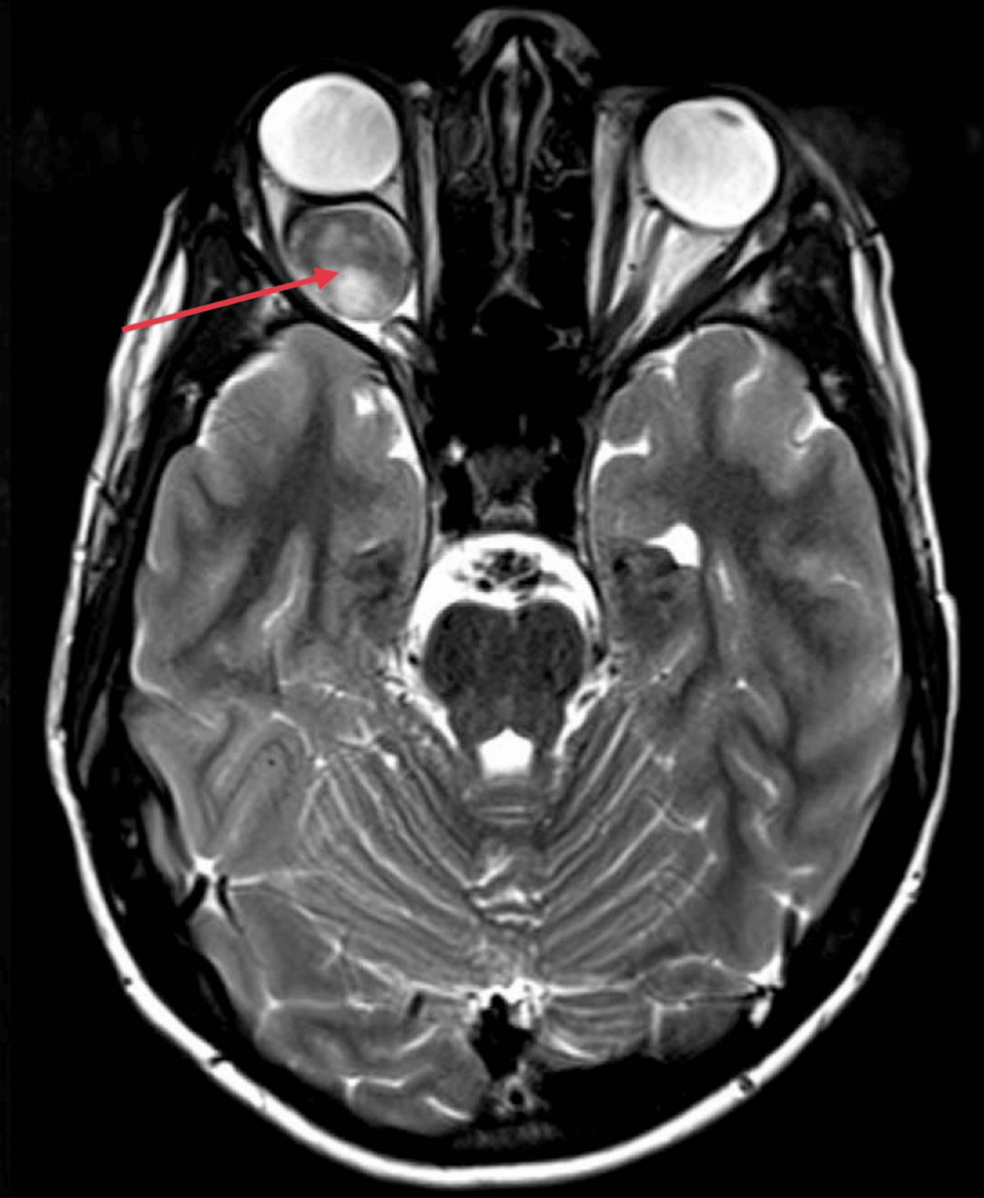
Fusiform enlargement of the intraorbital optic nerve. Magnetic resonance imaging (MRI) shows a fusiform enlargement of the intraorbital segment of the optic nerve on the right side, with homogeneous intense contrast enhancement (indicated by red arrow). This enlargement caused a mass effect on the posterior surface of the right eye globe, resulting in proptosis.

Magnetic resonance imaging (MRI) of the brain and orbit was requested which shows diffuse fusiform enlargement of the intraorbital segment of the optic nerve on the right side, showing homogenous intense contrast enhancement, causing mass effect on the posterior surface of the right eye globe and resultant proptosis (Figure 2). The imaging feature was suggestive of the possibility of optic nerve glioma.

**Figure 3:**
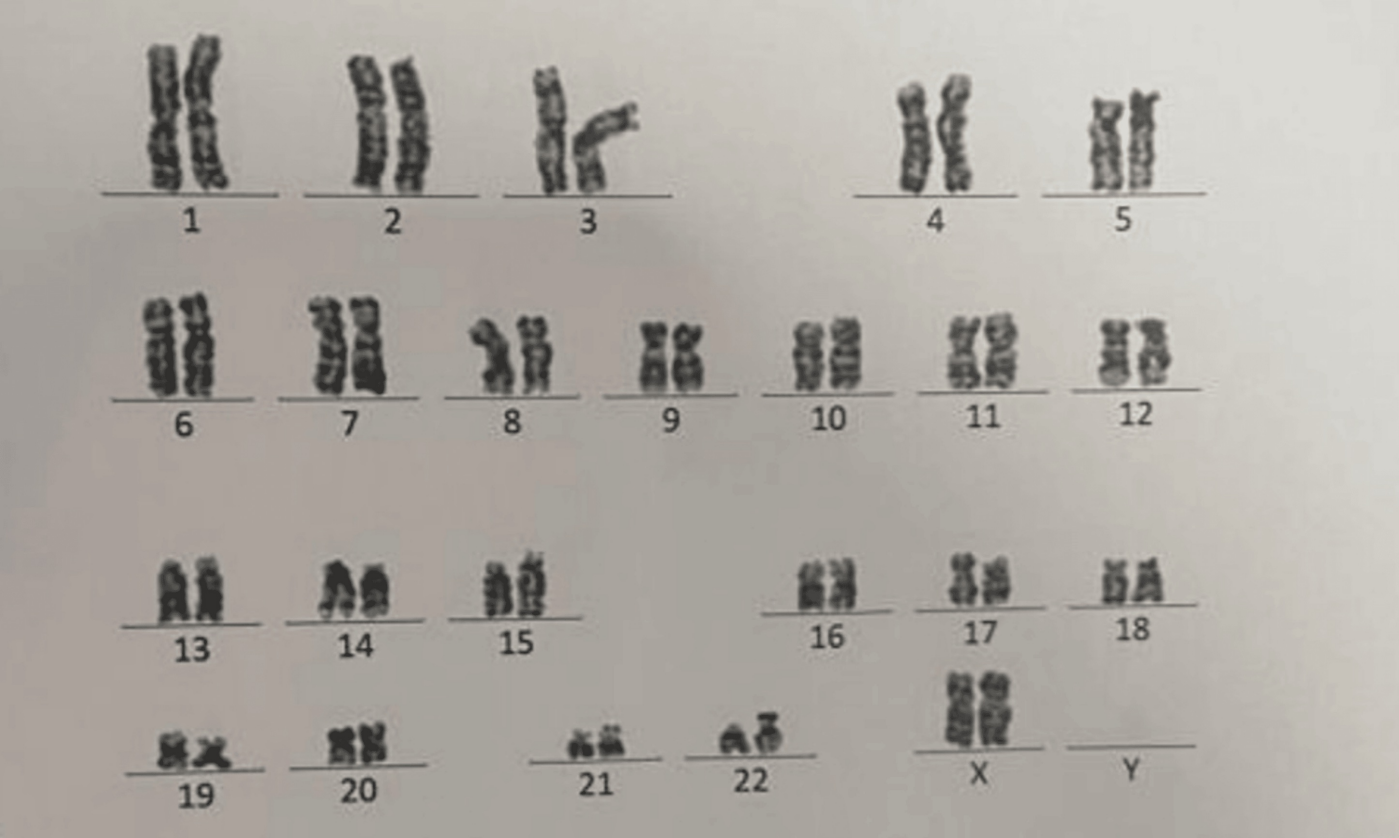
Genetic counseling and karyotyping. The genetic counseling and karyotyping report for the patient, parents, and siblings was considered normal based on the absence of chromosomal abnormalities and pathological findings.

Visual evoked potential (VEP) reports suggested absent waveform in the right eye and prolonged latency in the left eye suggestive of bilateral anterior optic pathway defect (right>left). Genetic counseling and karyotyping were performed on the parents, siblings, and patient, all of which revealed normal results (Figure 3).

**Figure 4:**
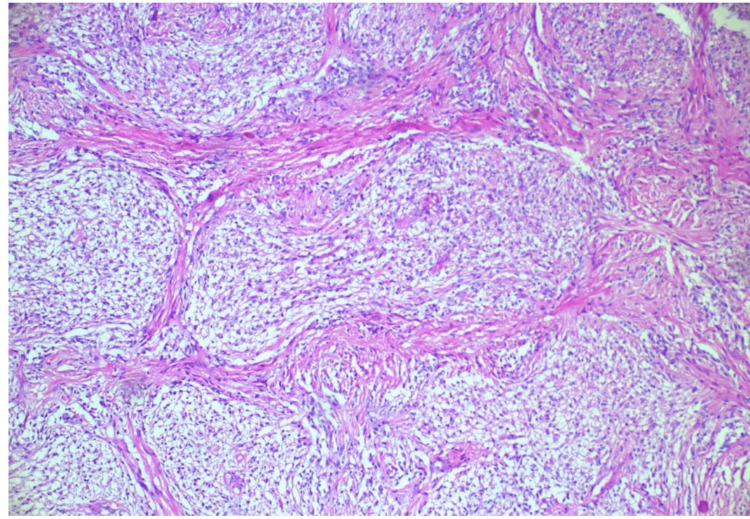
Cellular neoplasm with spindle-shaped nuclei and a biphasic pattern. Histopathological examination (HPE) shows cellular neoplasm composed of cells with spindle-shaped nuclei and biphasic pattern, consistent with a pilocytic appearance.

The patient’s parents were counseled regarding the nil visual prognosis in the right eye, and the patient was taken up for right optic nerve glioma excision via lateral orbitotomy by a team of oculoplasty and neurosurgery combined effort. Further, the excised optic nerve tumor mass was sent for histopathological examination (HPE) which showed cellular neoplasm composed of cells with spindle-shaped nuclei and appeared pilocytic (Figure 4).

Histopathological analysis shows cellular neoplasm with spindle-shaped nuclei and a biphasic pattern, consistent with pilocytic astrocytoma. No areas of necrosis, hemorrhage, cytologic atypia, or mitosis were seen. These features were consistent with optic nerve glioma (WHO grade I pilocytic astrocytoma). Post-operative optimum care was provided and the patient was discharged with consecutive follow-ups.

## Discussion

This case of a one-year-old child presenting with optic nerve glioma (ONG) without neurofibromatosis type 1 (NF1) underscores the importance of early diagnosis in pediatric patients with unilateral proptosis and vision loss. Typically, ONG is associated with NF1, and about 20-50% of ONGs present with this genetic condition, which often leads to a less aggressive clinical course [9]. However, this case did not show any signs of NF1, and the sporadic nature of the tumor required prompt intervention [10]. The differential diagnosis for this presentation includes optic neuritis, autosomal dominant optic atrophy (ADOA), and other intracranial neoplasms. MRI remains the gold standard for diagnosing ONGs, offering superior contrast resolution that allows for detailed imaging of the optic nerve and its surrounding structures [11]. In contrast, while CT can detect erosion or calcifications, it is less sensitive in early-stage optic nerve abnormalities [12]. The clinical management of ONGs in children varies based on the presence of NF1 and the progression of the tumor. For those with NF1, a conservative approach is typically employed, as the tumors often remain stable [13]. However, in the absence of NF1 and with signs of progressive disease, chemotherapy and radiation therapy are usually indicated [14]. Surgical intervention is considered when there is severe proptosis or a threat to intracranial structures, though complete excision is often not possible due to the risk of significant visual or neurological impairment [15]. This case highlights the critical need for multidisciplinary collaboration, involving ophthalmologists, radiologists, neurosurgeons, and oncologists, to ensure accurate diagnosis and timely intervention. The long-term prognosis of patients with ONG remains variable, and continuous monitoring with MRI is essential to assess treatment response and potential tumor progression [16]. Further studies are required to better understand the natural history of ONGs in non-NF1 patients, as well as the most effective therapeutic protocols for these sporadic cases [17].

## Conclusions

Optic nerve glioma (ONG) is the most common cause of unilateral proptosis in the pediatric population. Early diagnosis is crucial for optimizing management and improving prognosis. A multidisciplinary approach involving neurosurgeons, plastic surgeons, ophthalmologists, and radiologists is essential for delivering comprehensive care.

Timely intervention can lead to improved visual outcomes and effective management of proptosis. Neurosurgeons address surgical needs, while plastic surgeons manage cosmetic concerns. Ophthalmologists focus on preserving visual function, and radiologists provide critical imaging for accurate diagnosis and monitoring. Regular follow-up is vital to assess treatment response and detect potential tumor progression. Thus, a coordinated, multidisciplinary strategy is paramount to ensure favorable outcomes for children diagnosed with optic nerve glioma.
